# How Does Media Use Promote the Purchase of Private Medical Insurance? A Moderated Mediation Model

**DOI:** 10.3389/fpsyg.2022.894195

**Published:** 2022-06-10

**Authors:** Hao Shi, Lifei Gao, Guojun Wang

**Affiliations:** ^1^School of Insurance, University of International Business and Economics, Beijing, China; ^2^School of Economics, Beijing Technology and Business University, Beijing, China

**Keywords:** media use, private medical insurance, self-rated health status, cognitive ability, moderated mediation model

## Abstract

Various information media (such as TV and the Internet) have become the main channels through which for people to obtain information. Previous studies showed that media use influences the purchase of private medical insurance; however, research on its internal influence mechanism is still relatively weak. Using data from the Chinese General Social Survey 2017, this study constructed a moderated mediation model to analyze the mechanism of the influence of media use on the purchase of private medical insurance. Individuals’ self-rated health status was used as a mediator and individual cognitive ability was used as a moderator. The results showed that self-rated health status played a partial mediating role and individual cognitive ability played a negative moderating role in the direct path between media use and the purchase of private medical insurance. Furthermore, in the indirect path, individual cognitive ability negatively moderated the impact of media use on self-rated health status.

## Introduction

With society entering the information development stage, the media has become an indispensable part of our lives. Especially during the COVID-19 pandemic, the Internet has become our primary channel of news and health knowledge, and more and more people choose online healthcare platforms for medical treatment. Moreover, studies showed that when people watch movies about health risks, they learn the importance of private medical insurance through some online film and television review platforms, which promote individuals to purchase private medical insurance (Gao et al., 2022). When people learn about health and risk through the media, it is worth noting whether this news about risk events and health can increase people’s risk awareness and prompt them to take effective risk diversification measures (such as purchasing private medical insurance). In China, medical insurance includes social medical insurance schemes and private medical insurance. The social medical insurance schemes are funded by the government and aim to relieve the residents’ financial burden caused by illness, but they cannot fully compensate for medical expenses and have certain restrictions (Ramesh et al., 2014). Private medical insurance requires people to pay their own premiums, but it can provide the insured with out-of-pocket compensation for medical expenses (the medical expenses that the insured still needs to bear after the reimbursement of social medical insurance). Private medical insurance is also one of the effective means of risk diversification and sharing, which can affect the application of the health security system (Yip et al., 2012). However, since the launch of private medical insurance in 1998, the market demand for private medical insurance in China has been relatively low (Liu et al., 2018). Therefore, the factors influencing demand for private medical insurance deserve attention.

Some studies found that consumers will purchase private insurance only after they meet the basic consumption needs of life, and the possibility of purchasing private insurance increases with the increase in individual or family income (Showers and Shotick, 1994; Ying et al., 2007; Corman et al., 2009). A series of empirical studies about health economics suggested that residents with better health status who do not smoke and are not overweight are more likely to purchase private medical insurance (Jerant et al., 2012; Buchmueller et al., 2013). Additionally, an individual’s education level, employment status, employer type, and social medical insurance status have also been confirmed to affect the purchase of private medical insurance (Wan et al., 2020). Up to date, there hasn’t been much research regarding the effect of media use on purchasing private medical insurance. Their findings are mainly as follows: more information about insurance can be obtained when people use the media (Lee, 2012), thus improving their insurance literacy and enhancing their understanding of insurance (Browne and Kim, 1993). On the other hand, insurance companies can cooperate with media companies to popularize their insurance products among consumers through big data analysis and achieve more efficient sales (Brown and Goolsbee, 2000).

Are there other influencing mechanisms? Some studies have found that media use can affect an individual’s self-rated health status (Lohaus et al., 2005; Bundorf et al., 2006; Fenton and Panay, 2013). In the age of the low development level of the information media, people often seek advice about health from their friends or family who may also have a low level of health literacy (Loewenstein et al., 2013). Nowadays, the Internet can provide people with professional and comprehensive knowledge about health and healthcare online services. Those will help people develop healthy living habits and improve their health status. Furthermore, some studies found that their proactive behavior in seeking out health information may drive them to purchase private medical insurance (Furtado et al., 2016; Ndumbe-Eyoh and Mazzucco, 2016). However, to our knowledge, there is no study to explore the relationship between media use, health status, and private medical insurance. This study attempts to address these gaps by examining the mediating role of self-rated health status on the relationship between media use and the purchase of private medical insurance.

Additionally, studies revealed that people with varying cognitive abilities take different actions when given information about health and insurance (Lubinski, 2009; Fowler et al., 2017). In the process of using the media, individuals with different cognitive abilities judge the credibility of information differently when the media releases the same information (Lubinski, 2009). Previous studies suggested that Internet use has an impact on the health of the elderly, and the whole process is negatively moderated by individual cognitive ability (Wang et al., 2020). Therefore, both theoretically and empirically, it is necessary to explore whether individual cognitive ability plays a moderating role in the direct and indirect paths of the impact of media use on purchasing private medical insurance.

Overall, the core issue of this study included two aspects: (1) whether the mediating effect of self-rated health status was significant and (2) whether the moderating effect of cognitive ability was significant. Using data from the Chinese General Social Survey 2017, this study constructed a moderated mediation model to study the core issue. The results showed that self-rated health status played a mediating role between media use and the purchase of private medical insurance. Cognitive ability played a negative moderating role in both direct and indirect paths. This study enlarges the scope of the research of private medical insurance’s demand to explicitly include both self-rated health status and cognitive ability simultaneously. The research may give practitioners an increased understanding of how media use affects the purchase of private medical insurance, which can then be used to help insurance companies and the government spread knowledge about insurance, especially in the emerging area of big data analysis. The theoretical contribution of this paper is to further enrich the analysis of the mechanism of the effect of media use on the purchase of private medical insurance.

The remaining sections of this paper are as follows: Section “Theoretical Background and Hypothesis Development” introduces the theoretical background and hypothesis development. Section “Data, Variables, and Methods” explains the variable sources and the estimation method. Section “Findings” reports the findings. In Section “Robustness Check,” the robustness of the regression results is presented. Section “Discussion” presents a discussion. The final section is the conclusion of this paper.

## Theoretical Background and Hypothesis Development

### Media Use and the Purchase of Private Medical Insurance

The theory of planned behavior (TPB) (AJZEN, 1991) and the health-belief model (HBM) (Rosenstock et al., 1988) have become theoretical frameworks for the analysis of health-related behavior (McEachan et al., 2011; Huang et al., 2020), and have been supported by some empirical research in the field of consumer behavior (Amaro and Duarte, 2015). The TPB held that individuals’ behavior is decided by their intentions, which is mainly influenced by their attitude toward the behavior, subjective norm, and perceived behavioral control (AJZEN, 1991). The HBM emphasized the effect of individual health beliefs on future preventive behavior (Rosenstock et al., 1988). Both theories use individual-level approaches to predict health and risk transfer behaviors (Gerend and Shepherd, 2012), and these decisions are made by individuals in a deliberative process. Moreover, in the traditional theory of insurance demand, economists believed that consumers purchase insurance to transfer uncertain risks and receive corresponding economic compensation when risk events occur (Knight and Hyneman, 1921). Arrow (1963) proposed that risk avoiders are more willing to purchase insurance than risk seekers in the medical-care market based on three assumptions: that consumers are completely rational; the insurance market is a perfectly competitive market; and insurance payouts can fully cover economic losses. Since these three assumptions are obviously different from the actual market, the traditional theory of insurance demand has obvious limitations. With the development of behavioral economics, the modern theory of insurance demand proposed a decision-process on risk and policy analysis (Kunreuther et al., 1984). Based on the TPB, HBM, and modern insurance demand theory, we can infer that an individual could first identify the nature of risk associated with health and safety by collecting a large amount of information, then assess the potential loss caused by such risk, and finally decide to purchase insurance. If the level of information media development is low, the whole process of searching for information needs to consume a lot of time and economic cost. However, nowadays, information media (such as the Internet and TV) are highly developed, which helps consumers obtain risk-related information more conveniently than before and provides a more effective way for insurance companies to spread risk and insurance knowledge.

According to some studies, media use can positively affect the purchase of private medical insurance (Browne and Kim, 1993; Brown and Goolsbee, 2000; Manski, 2000; Beiseitov et al., 2004; Liu et al., 2014; Karaca-Mandic et al., 2017). First, from the perspective of insurance companies, the development of information media can increase the exposure of insurance companies’ advertising, thus promoting the purchase of private medical insurance to consumers (Brown and Goolsbee, 2000). In particular, the development of the Internet can help insurance companies utilize diversified advertising platforms and optimize the service process. From the perspective of consumers, they can purchase medical insurance online due to the development of Internet technology, which brings great convenience to consumers (Li and Shiu, 2012). Some empirical studies showed that media use can drive consumers to purchase private medical insurance by improving an individual’s trust in society and interaction frequency with others (Manski, 2000; Beiseitov et al., 2004; Liu et al., 2014). The influence of social trust and social interaction on the purchase of private medical insurance is mainly reflected in endogenous interaction, which can enhance the prevalence of financial behavior in the group (Manski, 2000). Moreover, media use can also improve an individual’s financial literacy, which can increase the possibility of purchasing private medical insurance (Pahlevan Sharif et al., 2020). Therefore, the first hypothesis is proposed in this study.

***Hypothesis 1.** Media use has a significant positive impact on the purchase of private medical insurance*.

### Self-Rated Health Status as a Mediator

The HBM believed that when individuals learn health and risk information, they will make expected utility judgments about unhealthy and risky behaviors (Rosenstock et al., 1988). When they develop a belief that a serious health problem or risky behavior makes them vulnerable, they could follow a particular health recommendation to reduce the consequences of that risk (Rosenstock et al., 1988; Gerend and Shepherd, 2012). Lots of researchers suggested that individuals who use the media to learn about risk and health insurance are often more concerned about their health status (Lambert and Loiselle, 2007; Atkinson et al., 2009). A key focus of this study, which is often overlooked, is that the relationship between media use and the purchase of private medical insurance may be mediated by the self-rated health status of individuals. Individuals use the media to obtain health-related information, including physical and mental health information, to judge their own health status and take actions to improve their health (Bundorf et al., 2006). Some studies on psychology suggested that the use of social media can relieve the psychological pain of some patients (Fenton and Panay, 2013; Ndumbe-Eyoh and Mazzucco, 2016). They can see the positive treatment results shared by doctors and other patients on social media (Zhang et al., 2020), which can relieve the psychological pressure of patients. It has also been discovered that young adolescents who use various types of media to obtain health information can ease their self-perceived pressure (Lohaus et al., 2005).

Furthermore, many empirical researches indicated that self-rated health status has a casual impact on the purchase of private medical insurance (Shmueli, 2001; Love and Smith, 2010; Atella et al., 2012). Individuals with a poor self-rated health status will incur more expected medical expenses, and they are more likely to purchase medical insurance. However, from the perspective of insurance companies, in order to prevent the problem of adverse selection, they often need a health assessment of the insured. As a result, individuals in poor health status will be rejected from purchasing private medical insurance, while individuals in better health status are likely to be insured by private medical insurance (Atella et al., 2012). Additionally, researchers found individuals who have a better self-rated health status are less likely to take risky actions, suggesting they tend to be risk averse and more likely to purchase private medical insurance (Doiron et al., 2008). In sum, this study predicted the second hypothesis that media use may drive individuals to purchase private medical insurance by affecting their self-rated health status.

***Hypothesis 2.** Self-rated health status plays a mediating role between media use and the purchase of private medical insurance*.

### Individual Cognitive Ability as a Moderator

Based on the TPB theory and the theory of reasoned action (TRA), cognitive self-regulation plays an important role in the whole process, from the generation of belief and motivation to some behaviors (Ajzen, 2015; Tomczyk et al., 2020). People with different cognitive abilities process information differently, hold different attitudes toward certain things, and finally take different actions. Social-cognitive theory suggested that individuals are likely to be misled by false information when they use the media, especially on the Internet, where anyone can post information. It is the key to identifying false information that individuals have better cognitive ability. Individuals’ cognitive ability was classified in terms of their ability to apprehend experience, educe relations, and correlates (Spearman, 1927). Previous studies concluded that, even under ideal conditions, the initial effects of false information cannot be simply eliminated by pointing out that it is incorrect, particularly for individuals with relatively weak cognitive ability (De keersmaecker and Roets, 2017). On the contrary, strong cognitive ability and good cognitive literacy enable individuals to better understand the true meaning of information so as to take corrective actions (Maitra, 2010).

**FIGURE 1:**
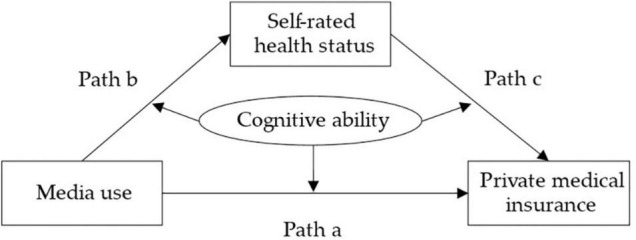
Conceptual framework.

In terms of health behavior, individuals with weak cognitive ability may overestimate their health status when they get some false information about health assessment (Lubinski, 2009; Fowler et al., 2017), and they continue to carry out some unhealthy life behaviors and do not take further measures to improve their health. Prior studies found the relationship between Internet use and health status of the elderly is negatively moderated by individual cognitive ability (Wang et al., 2020, 2021). Furthermore, based on financial behavioral theory, individuals need to browse news and obtain information to make financial decisions, which is usually the most beneficial to themselves (Lind et al., 2020; Pahlevan Sharif et al., 2020). However, the expected effect will not be achieved if they cannot make a reasonable judgment about the accuracy of the information. In addition, many insurance companies may exaggerate when selling in order to improve their sales performance. At this time, people with strong cognitive ability can understand the terms of insurance products well and will not purchase insurance. Therefore, individual cognitive ability is likely to negatively moderate the relationship between media use and the purchase of private medical insurance. This study proposed the third hypothesis and the fourth hypothesis, and drew the conceptual framework shown in Figure 1.

***Hypothesis 3.** Individual cognitive ability plays a negative moderating role in the direct path of media use on purchasing private medical insurance*.

***Hypothesis 4.** Individual cognitive ability plays a negative moderating role in the indirect path of media use on purchasing private medical insurance*.

## Data, Variables, and Methods

### Sample and Data Collection

The data were extracted from the Chinese General Social Survey 2017 (CGSS2017). The CGSS is the earliest continuous and comprehensive academic survey project in China. The first CGSS was launched by the Renmin University of China and the Hong Kong University of Science and Technology in 2003. The CGSS aims to systematically monitor the changing relationship between social structure and quality of life in China (Bian and Li, 2014). The CGSS2017 employed a stratified multi-stage probability proportional to size (PPS) sampling design and it consisted of three modules, namely, the core module, social network module, and family questionnaire module. These data contain essential information on the frequencies of media use, indicators of self-rated health and cognitive ability, and whether respondents purchased private medical insurance. The final analysis sample included 12,032 respondents with non-missing values in the variables of interest.

### Variables

The dependent variable, independent variable, mediator, moderator, and control variables used in this study are shown in Table 1.

**TABLE 1:** Variable definitions and descriptive statistics (*N* = 12,032).

Variable	Variable definition	Mean	Std. Dev.
**Dependent variable**			
Insurance	1 if the respondent has purchased private medical insurance, 0 if otherwise	0.12	0.32
**Independent variables**			
Traditional media use	From 1 to 5, the frequency of the respondent’s use of traditional media goes up	2.23	0.71
New media use	From 1 to 5, the frequency of the respondent’s use of new media goes up	2.25	1.24
**Mediator**			
SRH	From 1 to 5, the self-rated health status of the respondent gets better	3.63	0.88
**Moderator**			
Cog	From 1 to 5, the cognitive ability of the respondent gets stronger	2.45	0.78
**Control variables**			
Age	The age of the respondent	51.09	16.70
Age^2^	The square of the age	2889	1729
Gender	1 if the respondent is female, 0 if the respondent is male	0.53	0.50
Education	Education level of the respondent: “1 = junior college and below,” “2 = undergraduate,” and “3 = graduate and above”	5.16	3.27
Marital status	1 if the respondent is married, 0 if the respondent is unmarried	0.78	0.42
Family economic status	From 1 to 5, the family economic status of the respondent gets better	2.55	0.75
Social medical insurance	1 if the respondent has participated in social medical insurance, 0 if otherwise	0.92	0.27
Social endowment insurance	1 if the respondent has participated in social endowment insurance, 0 if otherwise	0.72	0.45

#### Dependent Variable

The dependent variable was measured using the question: “Do you have purchased private medical insurance?” The choice of “Yes” was coded as 1 and “No” was coded as 0.

#### Independent Variable

The core explanatory variable of this study is the frequency of media use of respondents, which included traditional media use and new media use (Hu and Li, 2019). Traditional media consisted of four items: newspapers, magazines, radio, and television. The respondents were asked how often they used these four kinds of media in the past year. The responses of each item were measured using a five-point Likert scale: “1 = never,” “2 = rarely,” “3 = sometimes,” “4 = often,” “5 = very frequent.” In this study, we used the average value of the four questions’ answers to constitute traditional media use, which was a continuous variable. New media use was measured using Internet and mobile customization messages. The respondents were asked to indicate how often they used those two media in the past year. The responses and coding were the same as the items of the traditional media use scale.

#### Mediator

Self-rated health as a mediator was measured using the respondents’ self-rated physical health and self-rated mental health (Wang and Geng, 2019). Regarding self-rated physical health, the respondents were asked, “How do you feel about your current physical health?” The responses were measured using a five-point Likert scale: “1 = very unhealthy,” “2 = relatively unhealthy,” “3 = average,” “4 = relatively healthy,” “5 = very healthy.” Regarding self-rated mental health, the respondents were asked, “In the past 4 weeks, how often did you feel depressed?” The responses were similarly measured using a five-point Likert scale: “1 = always,” “2 = often,” “3 = sometimes,” “4 = seldom,” “5 = never.” In this study, we took the average value of the sum of the self-rated physical and mental health status as a mediator, ranging from “1” to “5,” where the higher the value was, the better the respondents self-rated their health status.

#### Moderator

The moderator was individual cognitive ability. There is a clear agreement that cognitive ability is supported by mathematical, spatial, and verbal abilities (Lubinski, 2009). Furthermore, verbal ability plays an extremely important role in individual cognitive ability. There are four questions in the CGSS2017 that represent an individual’s cognitive ability (Wang et al., 2020): (a) “What do you think of your ability to listen to Mandarin?,” (b) “What do you think of your ability to speak Mandarin?,” (c) “What do you think of your English listening ability?,” (d) “What do you think of your English speaking ability?” The responses were measured using a five-point Likert scale: “1 = cannot speak at all,” “2 = relatively poor,” “3 = average,” “4 = relatively good,” “5 = very good.” This study obtained the moderator by adding the answers to these four questions together and then multiplying them by 0.25, and a higher value meant better individual cognitive ability.

#### Control Variables

Based on the previous literature review, the control variables selected in this study included age, the square of age, gender, education level, marital status, social health insurance, and social endowment insurance (Showers and Shotick, 1994; Ying et al., 2007; Corman et al., 2009; Lee, 2012; Buchmueller et al., 2013). Table 1 shows the definitions and assignments of these control variables. The impact of family income on the purchase of private insurance is greater than that of personal income (Corman et al., 2009); therefore, this study adopted family economic status as a control variable. Family economic status was coded as a five-item scale: “1 = well below average,” “2 = below average,” “3 = average,” “4 = above average,” “5 = well above average.” In addition, social insurance also has an impact on the purchase of private medical insurance (Ying et al., 2007); therefore, this study took social medical insurance and social endowment insurance as control variables.

### Methods

To analyze the mechanism of the influence of media use on the purchase of private medical insurance, the moderated mediation model was selected. We constructed Equations (1)–(3) based on the mediating effect model (Baron and Kenny, 1986), moderating effect model (Baron and Kenny, 1986), and moderated mediation model (Muller et al., 2005), respectively. Among the three equations, *Insurance*_*i*_ is the dependent variable, *Media*_*i*_ is the independent variable, *SRH*_*i*_ is the mediator, *Cog*_*i*_ is the moderator, and *X_i_* represents all variables that affect private medical insurance. *Media*_*i*_, *SRH*_*i*_, and *Cog*_*i*_ are all variables that were mean-centered because the process of mean-centering can reduce the problem of multicollinearity in regression equations and better explain the interpretation (Sinacore, 1991; Dawson, 2013). The interaction term between media use and cognitive ability is the product of media use and cognitive ability. The interaction term between self-rated health status and cognitive ability is the product of self-rated health status and cognitive ability. In addition, the mean-centering did not make any difference to the testing of the interaction term; the *p*-value for the interaction term and the subsequent interaction plot should be identical (Kromrey and Foster-Johonson, 1999; Dalal and Zickar, 2011). *Control*_*ij*_ indicates the control variables, including age, age^2^, gender, education, marital status, family economic status, social medical insurance, and social endowment insurance. α, β, and γ values are coefficients to be determined, while ε_*i*_, μ_*i*_, and ϵ_*i*_ are error terms.


$$Pr\left(Insurance_{i}=1|X_{i}\right)$$

$$=\Phi (\alpha_{0}+\alpha_{1}Media_{i}+\alpha_{2}Cog_{i}+\alpha_{3}Media_{i}*Cog_{i}$$

(1) $$\;+\sum \alpha_{j}Control_{i\,j}+\varepsilon_{i})$$

$$S\,R\,H_{i}=\beta_{0}+\beta_{1}\,M\,e\,d\,i\,a_{i}+\beta_{2}\,C\,o\,g_{i}+\beta_{3}\,M\,e\,d\,i\,a_{i}*C\,o\,g_{i}$$

(2) $$+\sum \beta_{j}\,C\,o\,n\,t\,r\,o\,l_{i\,j}+\mu_{i}$$

$$Pr\left(Insurance_{i}=1|X_{i}\right)=\Phi (\gamma_{0}+\gamma_{1}Media_{i}+\gamma_{2}Cog_{i}$$

$$\;+\gamma_{3}\,M\,e\,d\,i\,a_{i}*C\,o\,g_{i}+\gamma_{4}\,S\,R\,H_{i}+\gamma_{5}\,S\,R\,H_{i}*C\,o\,g_{i}$$

(3) $$\;+\sum \gamma_{j}Control_{i\,j}+\epsilon_{i})$$

Since the dependent variable is binary, Equations (1) and (3) used the probit model. When analyzing the mediating and moderating effects, the coefficient significance test of the probit model is the same as that of the OLS, but the coefficients cannot be directly used to calculate the effect size (Iacobucci, 2012). *SRH*_*i*_ is a continuous variable; therefore, Equation (2) adopted the OLS model. If the coefficient α_1_ is significant (*H_0_*: α_1_ = 0 is rejected), we conclude that media use significantly affects the purchase of private medical insurance. At the same time, if the coefficient α_3_ is significant (*H_0_*: α_3_ = 0 is rejected), we conclude that cognitive ability plays a moderating role in path a (Figure 1).

This study utilized the causal step test for mediating effects (Baron and Kenny, 1986). The causal step test requires that if β_1_ in Equation (2) is significant (*H_0_*: β_1_ = 0 is rejected) and γ_4_ in Equation (3) is significant (*H_0_*: γ_4_ = 0 is rejected), we show that the mediating effect exists. Moreover, if γ_1_ in Equation (3) is significant (*H_0_*: γ_1_ = 0 is rejected) at the same time, we get a partial mediating effect model, otherwise we get a full mediating effect model. Although the power of the causal step test method is low, the causal step test methods are very unlikely to commit a type I error (MacKinnon et al., 2002). This indicates that if the results of the casual step test are significant, the conclusion of the presence of the mediating effect can be obtained.

Assuming that the mediating effect is significant, β_3_ in Equation (2) or γ_5_ in Equation (3) is significant (*H_0_*: β_3_ = 0 or *H_0_*: γ_5_ = 0 is rejected), indicating that cognitive ability plays a moderating role in the indirect path (Edwards and Lambert, 2007). If β_3_ is significant, cognitive ability plays a moderating role in path b (Figure 1). Moreover, if γ_5_ is significant, cognitive ability plays a moderating role in path c (Figure 1).

## Findings

### Sample Descriptive Statistics

Table 1 shows the descriptive statistics of all variables used in this study. The total number of samples was 12,032 and 12% of respondents in the total sample had purchased private medical insurance. In the whole sample, the frequency of using new media was 2.25, which was higher than that of using traditional media (2.23), which was due to the development of the Internet in China in recent years (Li and Shiu, 2012). In terms of self-rated health status and cognitive ability, the average scores of self-rated health and cognitive ability in the total sample were 3.63 and 2.45, respectively. Furthermore, the proportion of respondents that participated in social medical insurance was over 90%, which showed that the social medical insurance schemes implemented by the Chinese government were very effective (Wu et al., 2018).

This study also conducted a statistical analysis according to the frequency of traditional media and new media use (Table 2). When the frequency of a respondent’s traditional media use exceeded the average of the total sample, they were assigned to the group named “high frequency of traditional media use.” Otherwise, they were assigned to the group named “low frequency of traditional media use.” A total of 5,445 samples with high-frequency use and 6,587 samples with low-frequency use of traditional media were obtained. The same method was applied to the division of the frequency of new media use. A total of 5,911 samples with high-frequency use and 6,121 samples with low-frequency use of new media were obtained. Table 2 shows that 15% of respondents with a high frequency of traditional media use had purchased private medical insurance, whereas only 8% of respondents with a low frequency of traditional media use had purchased private medical insurance. Individuals who used traditional media with a high frequency were more likely to purchase private medical insurance than those who used traditional media with a low frequency (Browne and Kim, 1993; Karaca-Mandic et al., 2017). When it came to new media use, the same result was obtained: 20% of the group with a high frequency of new media use had purchased private medical insurance, whereas only 4% of the group with a low frequency of new media use had purchased medical insurance, and the group with a high frequency of new media use was more likely to purchase private medical insurance (Browne and Kim, 1993; Ying et al., 2007).

**TABLE 2:** Descriptive statistics grouped by the frequency of traditional and new media use and self-rated health status.

	Traditional media use		New media use		Self-rated health status	
	High frequency of traditional media use (*n* = 5,445)	Low frequency of traditional media use (*n* = 6,587)	High frequency of new media use (*n* = 5,911)	Low frequency of new media use (*n* = 6,121)	Better self-rated health status (*n* = 6,063)	Poor self-rated health status (*n* = 5,969)
	
**Variable**	**Mean**	**Mean**	**Mean**	**Mean**	**Mean**	**Mean**
Insurance	0.15	0.08	0.20	0.04	0.15	0.08
Traditional media use	2.85	1.71	2.35	2.11	2.31	2.15
New media use	2.59	1.96	3.36	1.17	2.56	1.93
SRH	3.76	3.52	3.91	3.35	4.34	2.90
Cog	2.68	2.25	2.89	2.02	2.67	2.22
Age	51.10	51.08	41.04	60.80	47.11	55.13
Age^2^	2900	2880	1881	3862	2496	3288
Gender	0.49	0.56	0.51	0.55	0.50	0.56
Education	6.22	4.28	7.02	3.36	5.97	4.32
Marital status	0.79	0.77	0.76	0.79	0.79	0.77
Family economic status	2.70	2.43	2.71	2.39	2.72	2.38
Social medical insurance	0.94	0.91	0.92	0.92	0.92	0.93
Social endowment insurance	0.77	0.68	0.70	0.75	0.73	0.72

In terms of their self-rated health status, the respondents with a high frequency of traditional media use had an average self-rated health status score of 3.76, which was higher than the average score of 3.52 for those with a low frequency of traditional media use. Moreover, the average self-rated health score of the respondents with a high frequency of new media use was 3.91, which was also higher than the average self-rated health score of the respondents with a low frequency of new media use with 3.35. Table 2 also shows descriptive statistics grouped by self-rated health status. When the self-rated health status of a respondent exceeded the average of the total sample, they were assigned to the group named “better self-rated health status.” Otherwise, they were assigned to the group named “poor self-rated health status.” A total of 6,063 samples with a better self-rated health status and 5,969 samples with a poor self-rated health status were obtained. A total of 15% of the respondents with a better self-rated health status had purchased private medical insurance compared with just 8% of respondents with a poor self-rated health status. Individuals with a better self-rated health status were more likely to purchase private medical insurance (Cooper and Trivedi, 2012; Lee, 2012).

### Correlation Analysis

Correlations among dependent variable, independent variables, mediator, and moderator variables are listed in Table 3. According to Table 3, both traditional media use and new media use were significantly positively correlated with the purchase of private medical insurance. A significant positive correlation was found between media use and self-rated health status. In addition, there was a significant positive correlation between self-rated health status and the purchase of private medical insurance.

**TABLE 3:** Correlations among study variables.

Variable	Insurance	Traditional media use	New media use	SRH	Cog
Insurance	1				
Traditional media use	0.087***	1			
New media use	0.259***	0.213***	1		
SRH	0.281***	0.252***	0.573***	1	
Cog	0.130***	0.140***	0.314***	0.336***	1

***p < 0.01.

### Mediation Analysis

The regression results of mediating effect model of media use on the purchase of private medical insurance are listed in Table 4. Columns (1) and (4), respectively, report on the direct impact of traditional media use and new media use on the purchase of private medical insurance, namely, the test of Equation (1). Columns (1) and (4) show that the coefficients of traditional media use and new media use were 0.19 and 0.23, respectively, and both were significant at the 1% confidence level. This means that by increasing traditional media use and new media use by one scale, the probability of purchasing private medical insurance would increase by 3.09 and 3.65%, respectively. Thus, hypothesis 1 received support.

**TABLE 4:** Regression results of mediating effect model of media use on the purchase of private medical insurance.

	(1)	(2)	(3)	(4)	(5)	(6)

**Variable**	**Insurance**	**SRH**	**Insurance**	**Insurance**	**SRH**	**Insurance**
Traditional media use	0.19***	0.13***	0.18***			
	(0.02)	(0.01)	(0.02)			
New media use				0.23***	0.10***	0.23***
				(0.01)	(0.01)	(0.01)
SRH			0.09***			0.08***
			(0.02)			(0.02)
Age	0.03***	−0.03***	0.03***	0.03***	−0.03***	0.03***
	(0.01)	(0.00)	(0.01)	(0.01)	(0.00)	(0.01)
Age^2^	−0.00***	0.00***	−0.00***	−0.00***	0.00***	−0.00***
	(0.00)	(0.00)	(0.00)	(0.00)	(0.00)	(0.00)
Education level	0.43***	0.03	0.43***	0.38***	0.01	0.38***
	(0.04)	(0.02)	(0.04)	(0.04)	(0.02)	(0.04)
Marital status	−0.00	0.11***	−0.02	−0.02	0.11***	−0.03
	(0.05)	(0.02)	(0.05)	(0.05)	(0.02)	(0.05)
Gender	−0.01	−0.12***	0.00	−0.00	−0.12***	0.01
	(0.03)	(0.01)	(0.03)	(0.03)	(0.01)	(0.03)
Family economic status	0.24***	0.27***	0.22***	0.23***	0.27***	0.21***
	(0.02)	(0.01)	(0.02)	(0.02)	(0.01)	(0.02)
Social medical insurance	−0.24***	−0.11***	−0.23***	−0.22***	−0.10***	−0.22***
	(0.06)	(0.03)	(0.06)	(0.06)	(0.03)	(0.06)
Social endowment insurance	0.20***	0.08***	0.20***	0.16***	0.07***	0.16***
	(0.04)	(0.02)	(0.04)	(0.04)	(0.02)	(0.04)
Constant	−2.35***	0.53***	−2.39***	−2.67***	0.26***	−2.69***
	(0.18)	(0.08)	(0.18)	(0.18)	(0.08)	(0.18)
Observations	12,032	12,032	12,032	12,032	12,032	12,032
Adj *R*^2^			0.18	0.18		
Pseudo *R*^2^	0.11	0.13			0.12	0.14

The robust standard errors are in parentheses. ***p < 0.01.

The tests for the influence of the independent variables on the mediator are reported in columns (2) and (5), where the coefficients of traditional media use and new media use were both significantly positive. This showed that the more frequently media was used, the better the self-rated status was. Since the coefficients of traditional media use and new media use in columns (2) and (5) were significant, and the coefficients of self-rated health status in columns (3) and (6) were significant, we concluded that self-rated health status had a mediating effect between media use and the purchase of private medical insurance. Meanwhile, the coefficients of the independent variables in columns (3) and (6) were both not significantly equal to zero. Therefore, self-rated health status played a partially mediating role. This implied that media use had both a direct and indirect effect on purchasing private medical insurance. On the one hand, media use had a direct positive effect on the purchase of private medical insurance (path a in Figure 1). On the other hand, media use promoted the individual purchase of private medical insurance by improving individuals’ self-rated health status (path b and path c in Figure 1).

Moreover, using SPSS 26.0 Macro PROCESS (Hayes, 2013), we conducted a bootstrap procedure (number of bootstrap samples: 5,000) to analyze the direct and indirect effects of media use on the purchase of private medical insurance. The results are shown in Table 5. The direct and indirect effects of traditional media use on the purchase of private medical insurance were 0.33 (SE = 0.04, CI = [0.25, 0.41]) and 0.03 (SE = 0.01, CI = [0.02, 0.04]), respectively. For new media use, the direct and indirect effects were 0.41 (SE = 0.03, CI = [0.35, 0.47]) and 0.02 (SE = 0.01, CI = [0.01, 0.03]), respectively. The 95% confidence interval limits for these results did not contain zero, which was evidence that self-rated health status plays a partially mediating role between media use and the purchase of private medical insurance. Therefore, hypothesis 2 was accepted.

**TABLE 5:** Direct and indirect effects of media use on the purchase of private medical insurance.

Independent variables		Effect	Se	95% Confidence intervals	
				LLCI	ULCI
Traditional media use	Direct effect	0.33	0.04	0.25	0.41
	Indirect effect	0.03	0.01	0.02	0.04
New media use	Direct effect	0.41	0.03	0.35	0.47
	Indirect effect	0.02	0.01	0.01	0.03

*Bootstrap size = 5,000. Direct and indirect effects are on a log-odds metric.*

### Moderation Analysis

**FIGURE 2:**
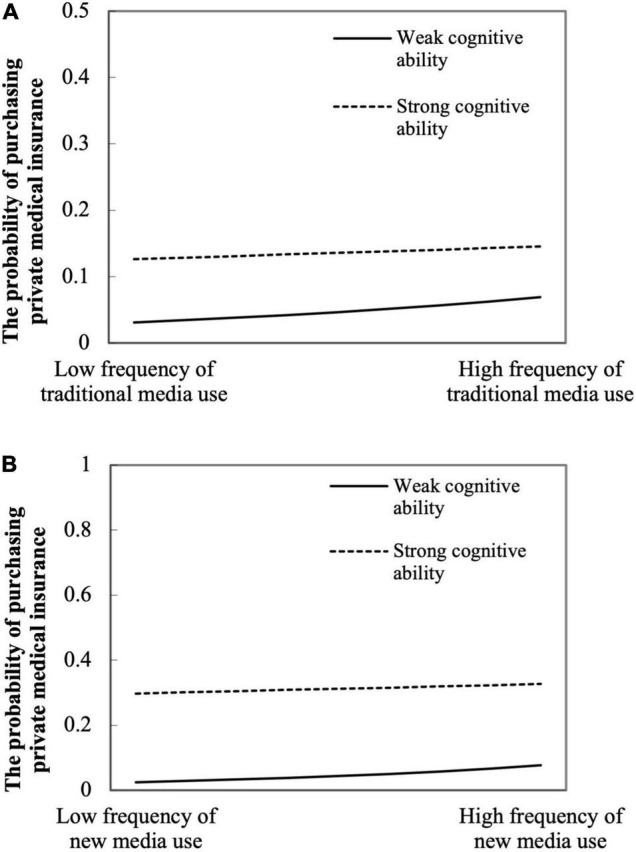
Moderating effect of cognitive ability on media use on the probability of purchasing private medical insurance. **(A)** Traditional media use; **(B)** new media use.

The coefficient of the interaction term between traditional media use and cognitive ability and the interaction term between new media use and cognitive ability in columns (1), (3), (4), and (6) of Table 6 were both significantly negative. This denoted that cognitive ability played a negative moderating role in the direct path (path a in Figure 1). We used the regression results in Table 6 to draw a graph (Figure 2) of the moderating effect of cognitive ability on path a (Figure 1; Dawson, 2013). Figure 2 shows that the relationship between media use and the probability of purchasing private medical insurance was always positive. Figure 2A demonstrates that the probability of purchasing private medical insurance rose more for people with weak cognitive ability (the solid line). This meant that strong cognitive ability weakened the effect of traditional media use on the likelihood of purchasing private medical insurance. Figure 2B implies that, although cognitive ability also had a negative moderating effect on the process of using new media to promote the purchase of insurance, the moderating effect was relatively small.

**TABLE 6:** Regression results of moderated mediation model of media use on the purchase of private medical insurance.

	(1)	(2)	(3)	(4)	(5)	(6)

**Variable**	**Insurance**	**SRH**	**Insurance**	**Insurance**	**SRH**	**Insurance**
Traditional media use	0.17***	0.09***	0.16***			
	(0.03)	(0.01)	(0.03)			
New media use				0.18***	0.07***	0.18***
				(0.02)	(0.01)	(0.02)
SRH			0.05**			0.04*
			(0.02)			(0.02)
Cog	0.40***	0.20***	0.40***	0.36***	0.20***	0.35***
	(0.03)	(0.01)	(0.03)	(0.03)	(0.01)	(0.03)
Traditionalmediause*Cog	−0.15***	−0.08***	−0.14***			
	(0.03)	(0.01)	(0.03)			
Newmediause*Cog				−0.04**	−0.07***	−0.04*
				(0.02)	(0.01)	(0.02)
SRH*Cog			−0.03			−0.01
			(0.03)			(0.03)
Control variables	Yes	Yes	Yes	Yes	Yes	Yes
Observations	12,032	12,032	12,032	12,032	12,032	12,032
Adj *R*^2^		0.20			0.20	
Pseudo *R*^2^	0.14		0.14	0.15		0.15

The robust standard errors are in parentheses. ***p < 0.01, **p < 0.05, *p < 0.1.

**FIGURE 3:**
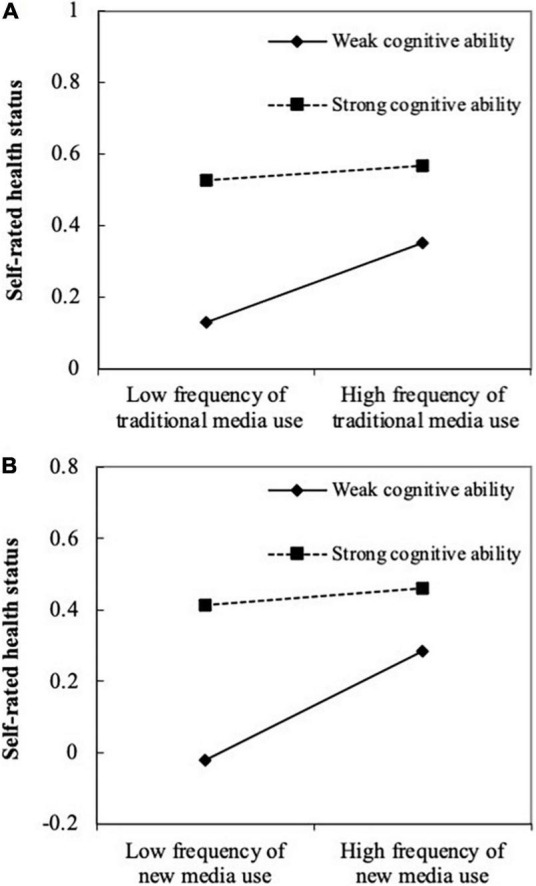
Moderating effect of cognitive ability on media use to self-rated health status. **(A)** Traditional media use; **(B)** new media use.

Table 6 also shows that the coefficient of the interaction term between traditional media use and cognitive ability and the interaction term between new media use and cognitive ability in columns (2) and (5) were both significantly negative. However, the coefficient of the interaction term between self-rated health status and cognitive ability was not significant in columns (3) and (6). This indicated that cognitive ability played a negative moderating role only for path b and not for path c (Figure 1) in the indirect path. Figure 3 shows the moderating effect of cognitive ability on path b (Figure 1; Dawson, 2013). Both traditional media use (Figure 3A) and new media use (Figure 3B) could improve an individual’s self-rated health status, but this effect was far greater for individuals with weak cognitive ability (the solid line) than for those with strong cognitive ability (the dotted line). Therefore, the results supported hypothesis 3 and hypothesis 4.

### Results of the Research Model

Hypothesis 1, hypothesis 2, hypothesis 3, and hypothesis 4 were supported, indicating that the moderated mediating effect model was significant. This study conducted a bootstrap procedure (number of bootstrap samples: 5,000) to further analyze the conditional direct and conditional indirect effects (Hayes, 2013). Table 7 reports the results. Regarding the conditional direct effect of traditional media use on purchasing medical insurance, it was 0.56 (SE = 0.08, CI = [0.40, 0.73]) when cognitive ability was weak, and it was 0.11 (SE = 0.05, CI = [0.02, 0.20]) when cognitive ability was strong. And the conditional indirect effect of traditional media use on purchasing private medical insurance *via* self-rated health status was statistically significant when cognitive ability was weak (−1 SD, indirect effect = 0.03, SE = 0.02, CI = [0.01, 0.06]) compared with when cognitive ability was strong (+ 1 SD, indirect effect = 0.01, SE = 0.01, CI = [−0.01, 0.01]). Regarding the conditional direct effect of new media use on purchasing private medical insurance, it was 0.43 (SE = 0.05, CI = [0.34, 0.53]) when cognitive ability was weak, and it was 0.25 (SE = 0.04, CI = [0.18, 0.32]) when cognitive ability was strong. The conditional indirect effect of new media use on purchasing private medical insurance *via* self-rated health status was statistically significant when cognitive ability was weak (−1 SD, indirect effect = 0.02, SE = 0.02, CI = [0.01, 0.04]) compared with when cognitive ability was strong (+ 1 SD, indirect effect = 0.01, SE = 0.01, CI = [−0.01, 0.01]).

**TABLE 7:** Conditional direct and conditional indirect effects of media use on the purchase of private medical insurance.

Independent variables		Level of cognitive ability	Effect	Se	95% Confidence intervals	
					LLCI	ULCI
Traditional media use	Conditional direct effect	−1 SD	0.56	0.08	0.40	0.73
		Mean	0.34	0.05	0.23	0.44
		+ 1 SD	0.11	0.05	0.02	0.20
	Conditional indirect effect	−1 SD	0.03	0.02	0.01	0.06
		Mean	0.02	0.01	0.01	0.02
		+ 1 SD	0.01	0.01	−0.01	0.01
New media use	Conditional direct effect	−1 SD	0.43	0.05	0.34	0.53
		Mean	0.34	0.03	0.28	0.41
		+ 1 SD	0.25	0.04	0.18	0.32
	Conditional indirect effect	−1 SD	0.02	0.02	0.01	0.04
		Mean	0.01	0.01	0.01	0.02
		+ 1 SD	0.01	0.01	−0.01	0.01

*Bootstrap size = 5,000. Direct and indirect effects are on a log-odds metric.*

**FIGURE 4:**
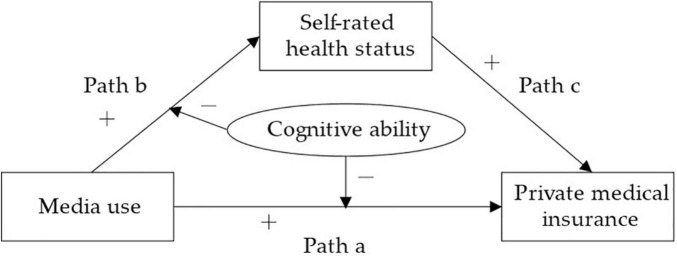
Influence mechanism diagram.

These results further supported that cognitive ability plays a negative moderating role in the direct path (path a in Figure 1) and the first half of the indirect path (path b in Figure 1) of media use on purchasing private medical insurance. After analyzing the mediation effect, the moderation effect, and the moderated mediating effect, this study gets the specific mechanism of action as shown in Figure 4.

## Robustness Check

### Replacement Estimation Method

Since the purchase of private medical insurance is a binary dependent variable, the probit model was adopted previously in the study, and the logit regression model was used for the presently discussed robustness check. The regression results are shown in Supplementary Table 1, and the conclusions were significant and robust. Media use had a positive impact on the purchase of private medical insurance, and the self-rated health status played a partially mediating role. Paths a and b in Figure 4 were negatively moderated by individual cognitive ability.

### Winsorizing

To alleviate the influence of outliers on parameter estimations, this study winsorized the independent variables at 2.5 and 97.5%. Supplementary Table 2 reports the regression results, which were also robust.

### Adding Control Variables

This study conducted a robustness test by increasing the control variable of social trust (Brown and Goolsbee, 2000). Social trust is an important factor in behavioral economics, where an individual’s trust in society will affect their judgment of social information, thus affecting their decision-making behavior. Social trust can effectively alleviate moral hazards and adverse selection problems when participating in medical insurance, which is beneficial for individuals when purchasing private medical insurance (Atim, 1999). The variable of social trust was composed of five scales. The higher the value was, the higher the level of trust the interviewees had in society. The regression results were significant and robust, as shown in Supplementary Table 3.

### Culling the Sample

Considering that the elderly use the Internet less (Bundorf et al., 2006), this study dropped the samples of respondents over 65 years old. The regression results are shown in Supplementary Table 4, where the regression results were still consistent with the previous ones after excluding people over 65 years old.

## Discussion

This study investigates the relationship between media use and the purchase of private medical insurance and their internal mechanism. As in hypothesis 1, the regression results of this study indicate that individuals’ use of traditional media or new media facilitated the purchase of private medical insurance. This finding is consistent with previous studies, which found that with an increasing frequency of media use, individuals are more likely to get insurance-related information (Cheong, 2007; Furtado et al., 2016), thus, are more likely to purchase insurance (Karaca-Mandic et al., 2017). Interestingly, we found that new media use promoted the purchase of private medical insurance more than traditional media use. The reason for this phenomenon could be that the Internet penetration rate in China has exceeded 70%, according to the China Statistical Yearbook 2021, and residents in some remote rural areas can use mobile phones to access the Internet at any time and from any location. In their daily lives, individuals spend far more time using new media than traditional media. In addition, when individuals see some news about health, risk, and insurance and want to purchase private medical insurance, they can do so online through the websites of insurance companies and insurance brokerage companies. When compared to purchasing insurance from an insurance agent in person, purchasing insurance online is more convenient and faster.

In addition, individual social trust, social interaction, and financial literacy improve with the increase in media use frequency, which drives them to purchase private medical insurance (Browne and Kim, 1993; Manski, 2000; Beiseitov et al., 2004; Liu et al., 2014). To further analyze the influence mechanism of media use on purchasing private medical insurance, we explored whether their relationship was mediated by individuals’ self-rated health status and whether the direct and indirect paths were moderated by individuals’ cognitive ability. We found that self-rated health status was played a partially mediating role between media use and the purchase of private medical insurance. Self-rated health status was positively correlated with media use and the purchase of private medical insurance. The direct and indirect paths from media use to the purchase of private medical insurance were negatively moderated by cognitive ability. Weak cognitive ability enhanced the direct effect of media use on purchasing private medical insurance and the indirect effect of media use on purchasing private medical insurance *via* self-rated health status.

### Mediating Role of Self-Rated Health Status

As in hypothesis 2, self-rated health status plays a partially mediating role and potential reasons might be attributed to the following. The media can provide individuals with information about physical and mental health and medical resources so that individuals can obtain rich health-related knowledge (Kim and Oh, 2012; Jiang and Street, 2017). People can also receive professional advice from some healthcare systems on issues such as eating habits, healthy lifestyles, and so on, to improve their physical quality of life. Compared with traditional media, the Internet can provide people with online expert consultation that is not limited by time and place, so that people can get a timely judgment of their health status, relieve their anxiety about their own body, and then obtain a better self-rating of their health status (Li et al., 2020). Furthermore, some studies have discovered that people who use the Internet to communicate with friends and build a harmonious social network have better mental health (Bessiere et al., 2010; Wellman et al., 2016). For some people who are often anxious, using the media to browse some entertainment news can divert their attention and reduce the symptoms of anxiety.

In addition, self-rated health status is often used as a measure of risk on the grounds (Hurd and McGarry, 1995; Idler and Benyamini, 1997). Studies have shown that people who have a better self-rated health status tend to do fewer risky things in life (Doiron et al., 2008). This type of person is risk averse and their desire for insurance is very high. Therefore, there may be a positive relationship between self-rated health status and the probability of purchasing private medical insurance. Moreover, individuals’ income is related to their health status, and those with a better self-rated health status are generally more likely to be employed and to have a sustained income than those with a poor self-rated health status. If they feel their future income is not sustainable and the premiums are not affordable, they may not choose to purchase private medical insurance (Yue and Zou, 2014). There is another reason for self-rated health status as a mediator. When people use the media to obtain information about health and insurance, they learn that people with poor health status will be rejected from purchasing private medical insurance. People with better health status worry about their future health status becoming poor and purchase private medical insurance in advance.

### Moderating Role of Cognitive Ability

As in hypothesis 3 and 4, this study found that individual cognitive ability negatively moderated the direct and indirect pathways of media use on purchasing private medical insurance. In the direct path, cognitive ability mitigated the positive effect of media use on purchasing private medical insurance. This phenomenon could be explained by the fact that people with strong cognitive ability were more cautious about insurance-related information (De keersmaecker and Roets, 2017). This leads to higher requirements for products and services for people with strong cognitive ability (Jhang et al., 2012). For the same insurance advertisement, they will be likely to find some flaws in private medical insurance due to their higher requirements. There are only a few private medical insurance products that can meet their requirements, which makes it more difficult to promote the purchase of private health insurance among them. However, it is quite interesting that the positive effect of new media use on purchasing private medical insurance is less negatively moderated by cognitive ability than traditional media use. This may be because, compared with traditional media, one of the advantages of new media is that people can search for relevant information at will, so that people are less affected by particular information. Thus, people can have a more comprehensive understanding of insurance and risk.

Meanwhile, the first half (path b in Figure 4) of the indirect effect was negatively moderated by cognitive ability. This was because people who had weak cognitive ability may overstate their health status (Tsendsuren et al., 2018). People with strong cognitive ability do not entirely trust media information about physical and mental health. They can examine the source and authenticity of the information and make more careful judgments about their health (De keersmaecker and Roets, 2017). Additionally, individuals who had strong cognitive ability were less optimistic about the true state of their physical and mental health. Therefore, as the frequency of media use increased, the self-rated health status of individuals with strong cognitive ability increased less than that of individuals with weak cognitive ability. In addition, interestingly, although the coefficient of the interaction term between self-rated health status and cognitive ability was not statistically significant, the coefficient value was negative. This may also indicate that people with strong cognitive ability have higher requirements for private medical insurance products, which may inhibit their demand for private medical insurance to some extent.

### Limitations and Future Directions

Due to the limitation of data, this study still had some deficiencies. First, the variable of cognitive ability in this study only included individuals’ verbal abilities and excluded mathematical and spatial abilities, which means that the measurement of cognitive ability may not have been comprehensive enough. Second, the CGSS2017 only included private medical insurance, without indicators for private health insurance. Finally, this study only analyzed the mechanism of the influence of media use on whether residents purchased private medical insurance but did not analyze the influence of media use on private medical insurance premium expenditure. Future studies can explore the mechanism of the influence of media use on the purchase of private health insurance and premium expenditure.

## Conclusion and Implications

The implementation of the social medical insurance policies (UEBMIS, NCMS, and URBMIS) in China has been very effective, but the coverage rate for private medical insurance is still very low. This study aimed to better explore the factors affecting the demand for private medical insurance and improve the coverage of private medical insurance in China. Based on the data of CGSS2017, this study found that media use had a significant positive impact on the purchase of private medical insurance, which is consistent with the conclusions of some previous studies. However, this study innovatively took self-rated health status as a mediating variable and took cognitive ability as a moderating variable to construct a moderated mediation model. The regression results showed that both mediating and moderating effects were significant. Media use directly and indirectly promoted the purchase of private medical insurance among individuals by improving their self-rated health status. Moreover, cognitive ability played a negatively moderating role in the direct path and the first half (path b in Figure 4) of the indirect path.

The theoretical contribution of this paper is to study the influencing factors of the purchase of private medical insurance on the basis of the TPB and HBM theories. Moreover, CGSS data are used to supplement the empirical research of the TPB and HBM theories using a moderated mediation effect model. Furthermore, this study can help us to better understand how media use influences the purchase of private medical insurance and provides a theoretical reference for governments around the world to publicize insurance knowledge through the media. From the perspective of practical contribution, this study will also provide help for insurance companies to adjust their advertising strategies. With the development of Internet media, the frequency of residents using the Internet media is increasing. Insurance companies can cooperate with Internet media, such as TikTok, Twitter, and Facebook, to deliver accurate insurance advertisements using big data analysis.

## Data Availability Statement

Publicly available datasets were analyzed in this study. This data can be found here: http://cgss.ruc.edu.cn/.

## Author Contributions

HS: conceptualization, methodology, software, formal analysis, writing–original draft preparation, and visualization. LG: methodology, validation, formal analysis, and visualization. GW: validation and funding acquisition. All authors contributed to manuscript revision, read, and approved the submitted version.

## Conflict of Interest

The authors declare that the research was conducted in the absence of any commercial or financial relationships that could be construed as a potential conflict of interest.

## Publisher’s Note

All claims expressed in this article are solely those of the authors and do not necessarily represent those of their affiliated organizations, or those of the publisher, the editors and the reviewers. Any product that may be evaluated in this article, or claim that may be made by its manufacturer, is not guaranteed or endorsed by the publisher.
