# Supplementary material for: How Does Media Use Promote the Purchase of Private Medical Insurance? A Moderated Mediation Model
Source: Front Psychol. 2022 Jun 10;13:894195. doi: 10.3389/fpsyg.2022.894195 (PMC9226612; doi:10.3389/fpsyg.2022.894195)
Supplement: Supplementary file 1 [file Data_Sheet_1.docx]

**Table A1.** Regression results of media use on the purchase of private medical insurance (using the logit model).

|  | **(1)** | **(2)** | **(3)** | **(4)** | **(5)** | **(6)** |
| --- | --- | --- | --- | --- | --- | --- |
| **Variable** | **Insurance** | **SRH** | **Insurance** | **Insurance** | **SRH** | **Insurance** |
| Traditional media use | 0.35*** | 0.09*** | 0.34*** |  |  |  |
|  | (0.05) | (0.01) | (0.05) |  |  |  |
| New media use |  |  |  | 0.35*** | 0.07*** | 0.34*** |
|  |  |  |  | (0.03) | (0.01) | (0.03) |
| SRH |  |  | 0.13*** |  |  | 0.10** |
|  |  |  | (0.05) |  |  | (0.05) |
| Cog | 0.75*** | 0.20*** | 0.76*** | 0.71*** | 0.20*** | 0.71*** |
|  | (0.05) | (0.01) | (0.05) | (0.06) | (0.01) | (0.06) |
| $Traditional media use*Cog$ | −0.31*** | −0.08*** | −0.29*** |  |  |  |
|  | (0.05) | (0.01) | (0.05) |  |  |  |
| $New media use*Cog$ |  |  |  | −0.13*** | −0.07*** | −0.12*** |
|  |  |  |  | (0.04) | (0.01) | (0.04) |
| Control variables | Yes | Yes | Yes | Yes | Yes | Yes |
|  |  |  |  |  |  |  |
| Observations | 12,032 | 12,032 | 12,032 | 12,032 | 12,032 | 12,032 |
| Adj R^2^ |  | 0.20 |  |  | 0.20 |  |
| Pseudo R^2^ | 0.14 |  | 0.14 | 0.15 |  | 0.15 |

The robust standard errors are in parentheses. *** p < 0.01, ** p < 0.05, * p < 0.1.

**Table A2.** Regression results of media use on the purchase of private medical insurance (winsorizing).

|  | **(1)** | **(2)** | **(3)** | **(4)** | **(5)** | **(6)** |
| --- | --- | --- | --- | --- | --- | --- |
| **Variable** | **Insurance** | **SRH** | **Insurance** | **Insurance** | **SRH** | **Insurance** |
| Traditional media use | 0.17*** | 0.09*** | 0.16*** |  |  |  |
|  | (0.03) | (0.01) | (0.03) |  |  |  |
| New media use |  |  |  | 0.18*** | 0.07*** | 0.18*** |
|  |  |  |  | (0.02) | (0.01) | (0.02) |
| SRH |  |  | 0.05** |  |  | 0.04* |
|  |  |  | (0.02) |  |  | (0.02) |
| Cog | 0.40*** | 0.20*** | 0.40*** | 0.36*** | 0.20*** | 0.35*** |
|  | (0.03) | (0.01) | (0.03) | (0.03) | (0.01) | (0.03) |
| $Traditional media use*Cog$ | −0.15*** | −0.08*** | −0.14*** |  |  |  |
|  | (0.03) | (0.01) | (0.03) |  |  |  |
| $New media use*Cog$ |  |  |  | −0.04** | −0.07*** | −0.04* |
|  |  |  |  | (0.02) | (0.01) | (0.02) |
| Control variables | Yes | Yes | Yes | Yes | Yes | Yes |
|  |  |  |  |  |  |  |
| Observations | 12,032 | 12,032 | 12,032 | 12,032 | 12,032 | 12,032 |
| Adj R^2^ |  | 0.20 |  |  | 0.20 |  |
| Pseudo R^2^ | 0.14 |  | 0.14 | 0.15 |  | 0.15 |

The robust standard errors are in parentheses. *** p < 0.01, ** p < 0.05, * p < 0.1.

**Table A3.** Regression results of media use on the purchase of private medical insurance (adding control variables).

|  | **(1)** | **(2)** | **(3)** | **(4)** | **(5)** | **(6)** |
| --- | --- | --- | --- | --- | --- | --- |
| **Variable** | **Insurance** | **SRH** | **Insurance** | **Insurance** | **SRH** | **Insurance** |
| Traditional media use | 0.17*** | 0.09*** | 0.16*** |  |  |  |
|  | (0.03) | (0.01) | (0.03) |  |  |  |
| New media use |  |  |  | 0.18*** | 0.07*** | 0.18*** |
|  |  |  |  | (0.02) | (0.01) | (0.02) |
| SRH |  |  | 0.05** |  |  | 0.04* |
|  |  |  | (0.02) |  |  | (0.02) |
| Cog | 0.40*** | 0.20*** | 0.40*** | 0.35*** | 0.20*** | 0.35*** |
|  | (0.03) | (0.01) | (0.03) | (0.03) | (0.01) | (0.03) |
| $Traditional media use*Cog$ | −0.15*** | −0.08*** | −0.14*** |  |  |  |
|  | (0.03) | (0.01) | (0.03) |  |  |  |
| $New media use*Cog$ |  |  |  | −0.04** | −0.07*** | −0.04* |
|  |  |  |  | (0.02) | (0.01) | (0.02) |
| Control variables | Yes | Yes | Yes | Yes | Yes | Yes |
|  |  |  |  |  |  |  |
| Observations | 11,994 | 11,994 | 11,994 | 11,994 | 11,994 | 11,994 |
| Adj R^2^ |  | 0.20 |  |  | 0.20 |  |
| Pseudo R^2^ | 0.14 |  | 0.14 | 0.15 |  | 0.15 |

The robust standard errors are in parentheses. *** p < 0.01, ** p < 0.05, * p < 0.1.

**Table A4.** Regression results of media use on the purchase of private medical insurance (dropping the samples of respondents over 65 years old).

|  | **(1)** | **(2)** | **(3)** | **(4)** | **(5)** | **(6)** |
| --- | --- | --- | --- | --- | --- | --- |
| **Variable** | **Insurance** | **SRH** | **Insurance** | **Insurance** | **SRH** | **Insurance** |
| Traditional media use | 0.20*** | 0.09*** | 0.19*** |  |  |  |
|  | (0.03) | (0.01) | (0.03) |  |  |  |
| New media use |  |  |  | 0.20*** | 0.07*** | 0.19*** |
|  |  |  |  | (0.02) | (0.01) | (0.02) |
| SRH |  |  | 0.06** |  |  | 0.05* |
|  |  |  | (0.03) |  |  | (0.03) |
| Cog | 0.41*** | 0.19*** | 0.41*** | 0.38*** | 0.20*** | 0.37*** |
|  | (0.03) | (0.01) | (0.03) | (0.03) | (0.02) | (0.04) |
| $Traditional media use*Cog$ | −0.16*** | −0.08*** | −0.15*** |  |  |  |
|  | (0.03) | (0.02) | (0.03) |  |  |  |
| $New media use*Cog$ |  |  |  | −0.06*** | −0.07*** | −0.05** |
|  |  |  |  | (0.02) | (0.01) | (0.02) |
| Control variables | Yes | Yes | Yes | Yes | Yes | Yes |
|  |  |  |  |  |  |  |
| Observations | 9193 | 9193 | 9193 | 9193 | 9193 | 9193 |
| Adj R^2^ |  | 0.18 |  |  | 0.18 |  |
| Pseudo R^2^ | 0.12 |  | 0.12 | 0.13 |  | 0.13 |

The robust standard errors are in parentheses. *** p < 0.01, ** p < 0.05, * p < 0.1.
